# Reconstituting the dynamics of endothelial cells and fibroblasts in wound closure

**DOI:** 10.1063/5.0028651

**Published:** 2021-01-19

**Authors:** Juliann B. Tefft, Christopher S. Chen, Jeroen Eyckmans

**Affiliations:** 1 The Biological Design Center and Department of Biomedical Engineering, Boston University, Boston, Massachusetts 02215, USA; 2 The Wyss Institute for Biologically Inspired Engineering, Harvard University, Boston, Massachusetts 02115, USA

## Abstract

The formation of healthy vascularized granulation tissue is essential for rapid wound closure and the prevention of chronic wounds in humans, yet how endothelial cells and fibroblasts coordinate during this process has been difficult to study. Here, we have developed an *in vitro* system that reveals how human endothelial and stromal cells in a 3D matrix respond during wound healing and granulation tissue formation. By creating incisions in engineered cultures composed of human umbilical vein endothelial cells and human lung fibroblasts embedded within a 3D matrix, we observed that these tissues are able to close the wound within approximately 4 days. Live tracking of cells during wound closure revealed that the process is mediated primarily by fibroblasts. The fibroblasts migrate circumferentially around the wound edge during early phases of healing, while contracting the wound. The fibroblast-derived matrix is, then, deposited into the void, facilitating fibroblast migration toward the wound center and filling of the void. Interestingly, the endothelial cells remain at the periphery of the wound rather than actively sprouting into the healing region to restore the vascular network. This study captures the dynamics of endothelial and fibroblast-mediated closure of three-dimensional wounds, which results in the repopulation of the wound with the cell-derived extracellular matrix representative of early granulation tissue, thus presenting a model for future studies to investigate factors regulating vascularized granulation tissue formation.

## INTRODUCTION

Wound healing is a critical process which progresses through tightly regulated phases and ultimately leads to repopulation of the wound with cells and extracellular matrix.^1^ A key aspect to this process involves the production of granulation tissue, a densely vascularized provisional tissue composed of endothelial cells, fibroblasts, and cell-derived matrix, which fills the wound area. This granulation tissue acts as the foundation for subsequent remodeling and healing.^2^ Poor vascularization of granulation tissue is often associated with slow healing or chronic wounds and is linked to certain risk factors such as diabetes, impaired blood flow in arteries or veins, or ischemic disease.^3,4^ Alterations in endothelial cells, fibroblasts, and wound matrix have been observed in chronic ulcers;^2^ however, the impact of these changes on healing progression is not fully understood, in part because the mechanisms by which fibroblasts and endothelial cells coordinate normal tissue closure and the assembly of vascularized granulation tissue remain elusive.

Recent studies of vascular morphogenesis have revealed an essential role for fibroblasts in the formation and maintenance of 3D vascular networks.^5–9^ This body of work has shown that fibroblasts are essential to the process of vascular network formation and stability^6^ due to their secretion of soluble factors^7,9^ and matrix components.^8^ Given the ability of fibroblasts to support vascular network formation in 3D gels *in vitro*, we sought to investigate whether fibroblasts contribute to the process of endothelial cell ingrowth and repopulation of the newly formed granulation tissue.

In this study, we capture the dynamic interactions of endothelial vessels and fibroblasts within a 3D matrix in the context of wound closure using a humanized *in vitro* model of vascularized wound healing and granulation tissue formation. This model utilizes human lung fibroblasts (HLFs) and human umbilical vein endothelial cells (HUVECs), which form spontaneous vascular networks when embedded in a fibrin gel. We injure these 3D vascularized tissues with incision wounds and use confocal microscopy of the live tissues over time to track the contributions of endothelial cells and fibroblasts toward the healing process. In agreement with our understanding of human healing *in vivo*, we demonstrate that fibroblasts serve the essential roles of wound contraction and of establishing the extracellular matrix within granulation tissue. Notably, in opposition to the rapid endothelial migration observed in traditional 2D scratch assays, our model demonstrates that only by following the initial wound closure by fibroblasts, can endothelial cells enter the healing region. In fact, even after closure, the endothelial cells remain primarily at the edges of the healed region rather than restoring the vascular network, thus adding a new perspective to our understanding of blood vessel dynamics in early stages of healing.

## RESULTS

### A platform to investigate the cooperation of endothelial cells and fibroblasts in wound healing and early granulation tissue formation

**FIG. 1.**
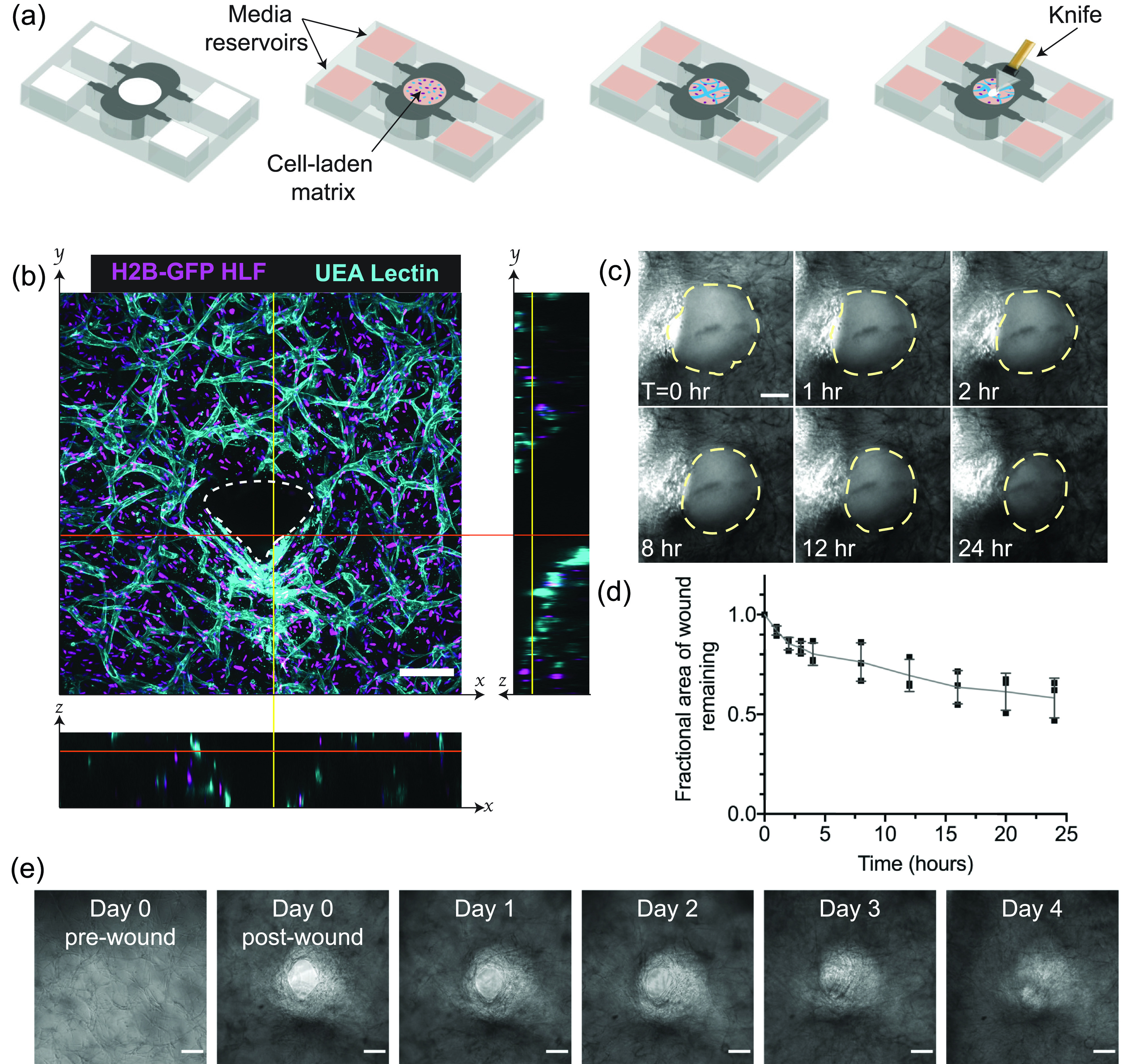
*In vitro* capillary beds can be injured and will heal in 3D over time. (a) Scheme for biomimetic vascularized wound healing. Endothelial cells and fibroblasts suspended within a fibrin gel are added to a PDMS mold and cultured for 3 days to develop a 3D capillary network. The devices are, then, wounded with a diamond dissection knife and imaged over time to track wound healing. (b) A wound in vascularized tissue, one day after wounding. The image is a z-projection of a 200 *μ*m confocal stack, with cross sectional views to indicate the wound depth. White dotted lines in cross sections indicate the wound borders. The scale bar is 150 *μ*m. (c) Brightfield image time-lapse over 24 h, with wound borders outlined with yellow dotted lines. The scale bar is 150 *μ*m. (d) Quantification of the reduction of the wound area over the 24 h period. Error bars represent mean ± STD (n = 3). (e) Brightfield images of tissues over the course of 4 days. The scale bar is 150 *μ*m.

In order to look at the early stages of wound healing in vascularized tissues, we formed vascularized tissues within a PDMS mold by mixing human umbilical endothelial cells (HUVECs) and human lung fibroblasts (HLFs) into a 3D collagen and fibrin composite gel [Fig. 1(a)]. The coculture of these two cell types within a 3D gel resulted in capillary network formation via vasculogenesis within 3 days [Fig. 1(a)] with an approximate tissue thickness of 0.5 mm. The PDMS mold used in this study was adapted from a previously published platform^8^ to enable the formation of vascular networks without fluid flow and to allow for tissue injury by uncovering the central gel region. We utilized a composite gel of 0.4 mg/ml collagen I and 2.5 mg/ml fibrinogen because these composite gels have been shown to retain the robust vasculogenesis, which is observed in pure fibrin gels,^10^ while also incorporating collagen I, a fibrous matrix molecule that imparts strength to mature tissue.^11^ The composite bulk gel had a storage modulus of 60.97 ± 9.57 Pa and a loss modulus of 6.47 ± 1.41 Pa (Fig. 1 in the supplementary material). Previous work has shown that similar vasculogenic tissues do get stiffer over time,^8,12^ with this increasing stiffness dependent largely on the presence of fibroblasts within the tissue.^8^

After three days of culture, these tissues were cut using a diamond dissection knife, yielding a full-thickness wound through the tissue [Figs. 1(a) and 1(b)]. Visualization of these vascularized tissues immediately post-injury revealed that the wounds did not fully close over the first 24 h, although the wound area was reduced to 69.4 ± 8.0% of the original wound area [Figs. 1(c) and 1(d); Movie 1 in the supplementary material]. Over the course of 4 days however, the wounds in these tissues did heal. Despite relatively stagnant wound edges between 1 and 2 days, cell migration into the wound was visible by day 3, with full closure achieved by day 4 [Fig. 1(e)].

### Wound closure is dependent on the presence of fibroblasts in the tissue

**FIG. 2.**
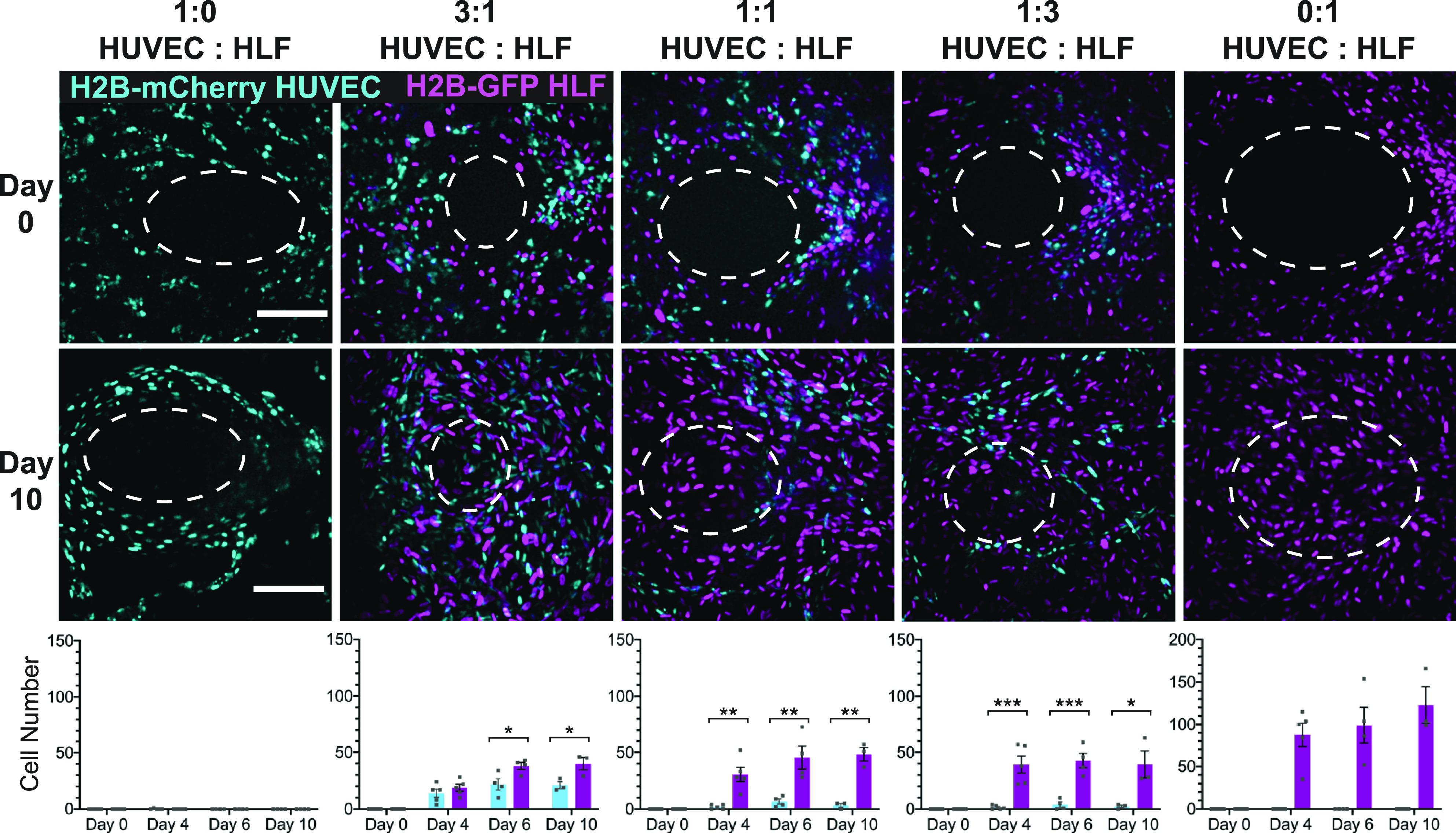
Wound closure is dependent on the presence of fibroblasts in the tissue. (Top) Projections of a confocal stack of images of the original wound area (day 0) for tissues with constant cell density but varying ratios of endothelial cells and fibroblasts. Ellipse outlines the original wound area. (Middle) Z-projections of the confocal stack of images taken after 10 days of healing, with the same ellipse overlay. (Bottom) Quantification of the number of H2B-GFP fibroblasts or H2B-mCherry HUVECs migrating into the wound over time tissues with varying starting cell ratios. The scale bar is 150 *μ*m. Error bars represent mean ± SEM (n = 3) with 1–2 devices per condition per experiment. The significance was determined using Student's t-test; *= p < 0.05, ** = p < 0.01, and *** = p < 0.001.

Having characterized the overall wound healing dynamics of this model, we were, then, interested in the relative contributions of endothelial cells and fibroblasts during healing. To achieve this, tissues were formed with constant cell density and five different ratios of endothelial cells to fibroblasts. After wounding, the healing of these tissues was tracked via live confocal microscopy once daily over the course of ten days. The average size of the initial injury was 0.0496 ± 0.019 mm^2^ with an average aspect ratio (major axis/minor axis) of 1.289 ± 0.237; however, there was no correlation between these two properties, R^2^ = 0.0003, suggesting that the wound geometry did not affect the size of the defect (Fig. 2 in the supplementary material). Cell tracking of live cells over the course of healing was enabled by labeling the primary cells with fluorescent histone 2B proteins. Tissues with endothelial cells alone did not exhibit cell migration into the void over the course of ten days (Fig. 2, left column). In contrast, tissues with fibroblasts alone displayed rapid cell migration into the wounded area (Fig. 2, right column), with full-thickness wound closure as early as by day two [Fig. 4(a)]. Tissues with intermediate ratios of endothelial cells and fibroblasts showed some endothelial migration into the healing area, with the number of cells migrating correlating with the starting number of endothelial cells present within the tissues (Fig. 2, center columns). In tissues of all intermediate cell ratios, by day six, there were significantly more HLFs than HUVECs within the 3D healing region, regardless of the starting density of endothelial cells within the bulk tissue (Fig. 2, bottom row, center columns). This evidence supports the hypothesis that fibroblasts are the primary driver of wound closure, with the emergence of endothelial cells within the healed area depending on the presence of the fibroblasts.

### Fibroblasts migrate tangentially to the wound during contraction, while vessels fluctuate at the wound periphery

**FIG. 3.**
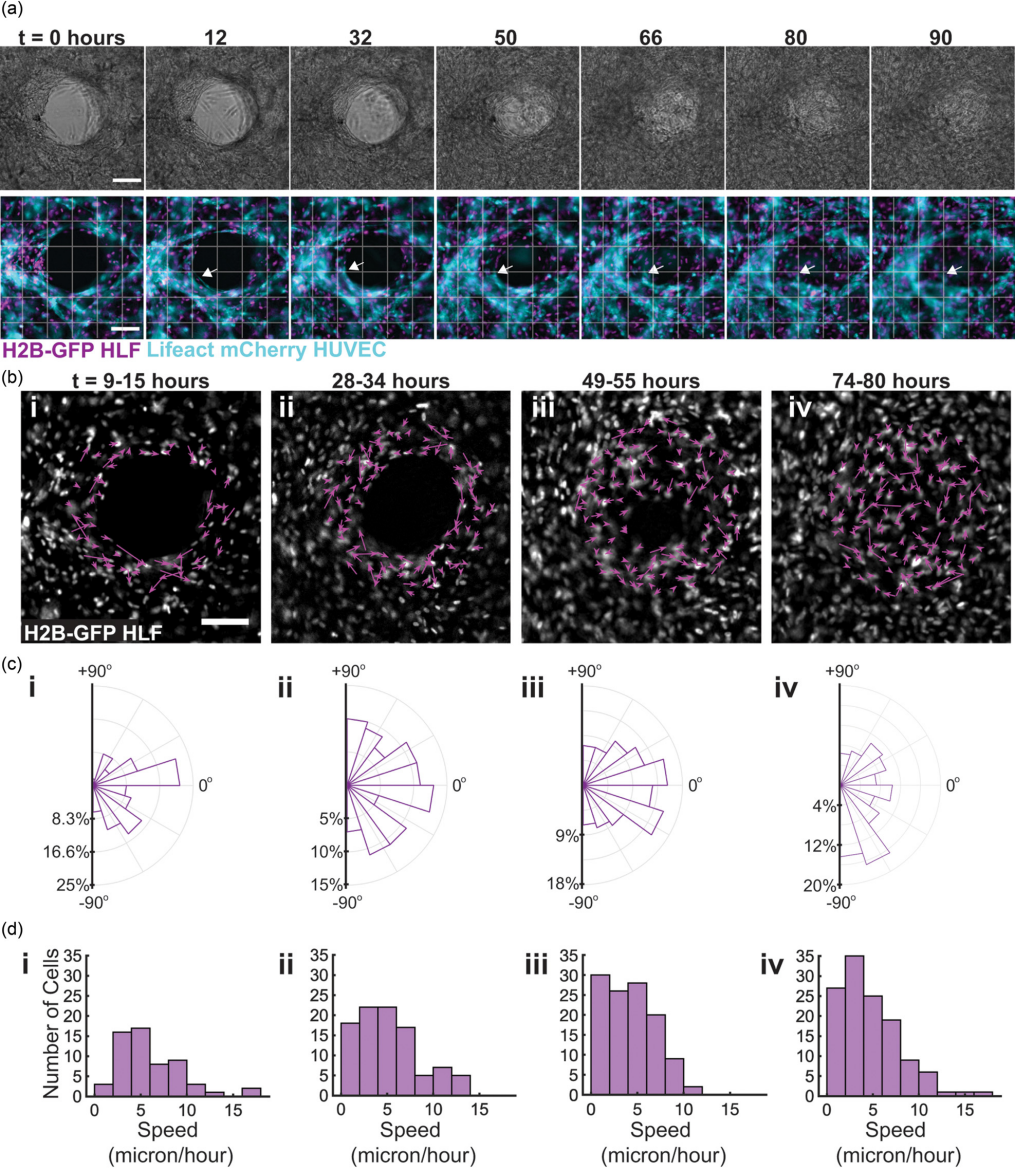
Fibroblasts migrate tangentially to the wound during contraction, while vessels fluctuate at the wound periphery. 96 h time lapse was performed to visualize healing progress over time in devices with a 1:1 ratio of HUVECs to HLFs. (a) Brightfield progression of healing over time (top). Fluorescent imaging of tissues over healing progression; the arrow indicates one vessel through the course of the time lapse (bottom). (b) Net displacement of fibroblast nuclei over 6 h segments of imaging, determined using the ImageJ Trackmate plugin. (c) Rose plots indicating the distribution of the direction of net displacement of fibroblasts for each imaging segment. (d) Histograms displaying the net velocities of individual fibroblasts over the course of each 6 h imaging segment. The scale bar is 150 *μ*m.

Although we found that the fibroblasts were essential to facilitate migration into the healing region, blood vessel ingrowth is an essential aspect of healing *in vivo*, and so, we were curious about the dynamics of the vessels during the closure process in our model. To achieve this, we prepared tissues with equal numbers of H2B-GFP HLFs and a live actin labeled LifeAct-mCherry HUVECs, injured the tissues, and monitored the healing process on an epi-fluorescence microscope in real time over the course of four days (Movies 2 and 3 in the supplementary material). Brightfield images of the wound show distinct wound margins through 30 h, but by 50 h, the fluorescence reveals the initiation of fibroblast migration into the wound void, with full gap closure by 80 h [Fig. 3(a)]. The migration of the fibroblasts and endothelial cells was tracked over the course of 90 h using the ImageJ plugin Trackmate, revealing rapid movement of fibroblasts around the wound edge and slower motion by endothelial cells (Fig. 3 in the supplementary material). The calculation of the net displacement of the cells over 6 h [Fig. 3(c)] indicated that the fibroblasts at the wound edge migrated circumferentially around the wound during the lag phase of healing [Figs. 3(b-i) and 3(b-ii)], until about 50 h, when cells began to close the void [Fig. 3(b-iii)]. Once closure was initiated, cells migrated toward the wound [Fig. 3(b-iii)], but once the gap was closed, the cellular motion became random [Fig. 3(b-iv)]. Rose plots indicate the angle of net displacement over the course of each 7 h image sequence [Fig. 3(b)].

During this progression, the endothelial vessels fluctuated behind the original wound edge throughout imaging [Fig. 4(a) in the supplementary material] and did not produce evident angiogenic sprouts toward the center region [Fig. 3(a), bottom]. Although the vessels remained at the periphery of the healing region over time, some vessels at the edge appeared to drift toward the center of the healing region over the course of imaging [Fig. 3(a), arrows]. Whether this process is as a consequence of the matrix contraction that occurs during the closure process or if it is an active process mediated by endothelial cells is not yet fully understood, but endothelial cells were primarily restricted to the borders of the healing region.

Cell velocities were calculated from the net displacement data and are plotted for the fibroblasts in Fig. 3(d) and for the endothelial cells in Fig. 4(c) in the supplementary material. The average velocities of fibroblasts over each imaging segment were 6.16 ± 3.8 *μ*m/h, 5.04 ± 3.35 *μ*m/h, 4.22 ± 2.7 *μ*m/h, and 4.73 ± 3.25 *μ*m/h for 9–15 h, 28–34 h, 49–55 h, and 74 h, respectively. The average velocity of the endothelial cells per imaging segment was 2.00 ± 1.15 *μ*m/h, 2.00 ± 1.55 *μ*m/h, 1.58 ± 1.27 *μ*m/h, and 1.59 *μ*m/h for each respective imaging segment. Comparison of the two groups via a Mann-Whitney U-test for non-Gaussian datasets revealed that the endothelial cells migrated at significantly lower velocities than the fibroblasts (P < 0.0001) for each respective 6 h imaging segment.

### Fibroblasts remodel the original fibrin matrix during the closure process

**FIG. 4.**
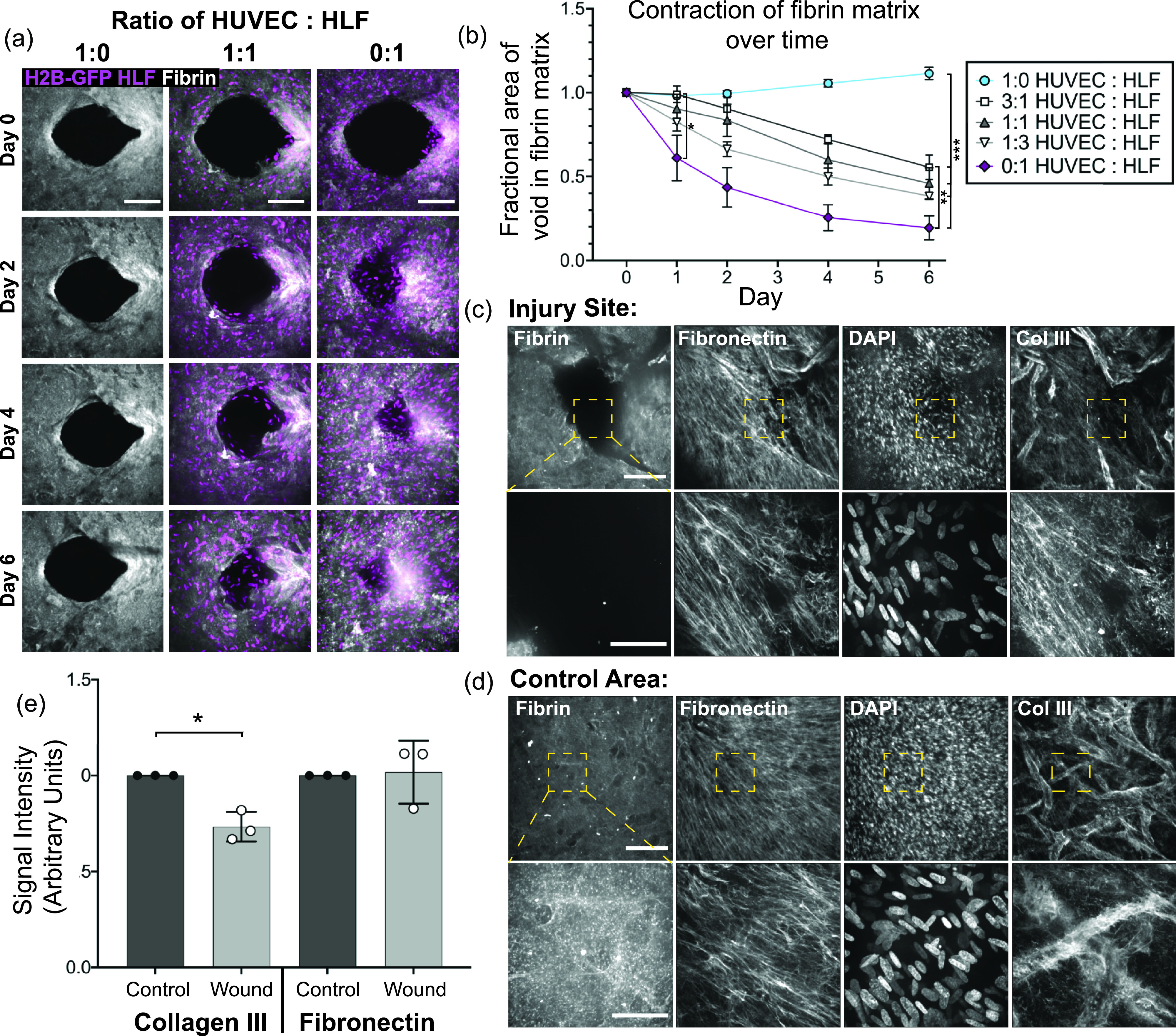
Fibroblasts remodel the original fibrin matrix during healing but deposit the provisional matrix into the wound area in order to close the gap. (a) Z-projection of the original Cy5-labeled fibrinogen matrix (gray) and HLF nuclei (magenta) over the course of healing. Tissues with HUVECs only (left), a 1:1 ratio of HUVECs to HLFs (middle), or HLFs only (right). The scale bar is 150 *μ*m. (b) Quantification of the void area remaining in the fibrin matrix over time for devices with varying ratios of HUVECs to HLFs. Error bars represent mean ± SEM, quantified from n = 4 tissues per condition. Data were compared using ANOVA with post hoc Tukey's test; *= p < 0.05; ** = p < 0.01; and *** = p < 0.001. (c) and (d) Immunofluorescent staining of fibronectin and collagen III in tissues with the pre-labeled Cy5-fibrin matrix six days post-wounding. Images at 10× (top row; the scale bar is 150 *μ*m) and 40× (bottom row; the scale bar is 50 *μ*m); the area imaged at higher magnification is indicated by the yellow box. (c) Images taken directly at the site of injury and (d) at a control site away from the wounded area. (e) Quantification of the fibronectin and collagen III staining intensity within the healed region or within the control tissue, quantified from 5 to 6 regions of interest in three different tissues from the 10× images as shown in (c) and (d). Data were compared with a paired t-test, * = p < 0.05.

Given that the fibroblasts drive gap closure in our model, we postulated that the fibroblasts were either remodeling the original fibrin matrix or depositing a new matrix to mediate gap closure. To address this question, we formed tissues with nuclear-labeled cells and added pre-labeled fluorescent fibrinogen with unlabeled fibrinogen in order to visualize both the fibrin matrix and cell migration over time. Images taken on consecutive days display a contraction of the original matrix over time, which was dependent on the number of fibroblasts present in the tissue [Figs. 4(a) and 4(b)]. Quantification of the area devoid in the fibrin matrix showed contraction of the native fibrin matrix in all cases except for the tissues composed of endothelial cells alone. Comparison of groups using ANOVA with post hoc Tukey's test showed that by day one, tissues with fibroblasts alone contracted the fibrin matrix significantly more than tissues with a 3:1 ratio of endothelial cells to fibroblasts (p < 0.0279), as well as compared to tissues with endothelial cells alone (p < 0.0322). By day six, more distinctions emerged, and the tissues with endothelial cells alone had contracted significantly less than all other conditions (p < 0.0002), and tissues with fibroblasts alone contracted more than tissues with 3:1 EC:HLF (p < 0.009). Increased contraction of the matrix, particularly during the first 24 h after injury (Fig. 5 in the supplemental material), showed a trend, which correlated with the number of fibroblasts in the tissue, but no significant differences existed between tissues formed with 50% or more fibroblasts [Fig. 4(b)]. While these data highlight a role for contraction of the native fibrin matrix in wound closure, in tissues composed of fibroblasts alone, cells were present in the three-dimensional void as early as by day two post-wounding, at which time the void in the native fibrin matrix was still highly visible [Fig. 4(a), right column]. These findings suggest that the native fibrin matrix did not provide the template for migrating fibroblasts to close the wound.

### Fibroblasts secrete provisional matrix molecules into the healing area for full closure

Given that cell migration into the 3D wound occurs without full closure of the gap remaining in the fibrin matrix, we were interested in whether other matrix molecules were deposited in this healed region and hypothesized that the cells would likely deposit matrix proteins known to be present in early granulation tissue. Immunofluorescent staining of tissues 6 days after wounding demonstrated the presence of both fibronectin and collagen III in the healing area [Fig. 4(c), top]. Imaging at higher magnification demonstrated the lack of fibrin in the healed area but the presence of fibronectin and collagen III fibers [Fig. 4(c), bottom]. Uninjured locations, which served as control areas, also contained fibronectin and collagen III fibers, but the collagen III fibers in the control tissue were localized preferentially to the basement membrane of endothelial vessels [Fig. 4(d)]. Quantification of the intensity of matrix staining in both the healed region and the control tissue also showed a lower intensity of collagen III in healed vs uninjured tissue (p < 0.0271), thus further corroborating our findings [Fig. 4(e)].

The collagen III staining showed increased intensity in the basement membrane of the blood vessels, allowing visualization of the location of blood vessels in these tissues. By looking at whether the vascular structures as visualized by collagen III staining were co-localized with the initial fibrin gel, further information was gathered about whether vessels were embedded within the original matrix or were sprouting into the new cell-derived matrix within the healed region. Overlay of the collagen III staining with the original fibrin indicated that the vessels were primarily present in the native, fibrin positive region of the tissue, although some vessels did enter the healing region at the border of the native and healed regions (Fig. 6 in the supplementary material). Together, these data indicate that the vessel structures are primarily restricted to the wound periphery, and movement of the vascular structures observed in Fig. 3 may be linked to tissue contraction of the initial matrix, which is observed over the course of healing.

## DISCUSSION

To our knowledge, this is the first demonstration of an *in vitro* wound closure model that requires the *de novo* formation of granulation tissue, providing a unique opportunity to observe the relative contributions of different cell types during early wound healing. Previous efforts to investigate fibroblast-endothelial interactions in wound healing have relied on animal models or 2D *in vitro* assays, neither of which have fully clarified their contribution to the healing process. Animal models capture the complexity of healing, but key differences between animal and human healing modes remain.^4,13^ For example, in mouse and rat wounds, contraction plays a major role, to the extent that formation of granulation tissue is not required for healing.^13^ To provide deeper insights into the healing process, cell culture models have been used extensively. The most widely used *in vitro* model of human wound healing is the scratch wound assay, in which a monolayer of cells is wounded by a mechanical scratch, which removes a section of cells, and the remaining cells migrate into the space to reform the monolayer.^14^ Scratch wound assays have often been used to investigate mechanisms of endothelial cell migration alone^15–18^ and also in the presence of fibroblasts^19^ but do not capture the complex cell-matrix interactions that are required for tissue closure during healing of a multilayered, extracellular matrix-rich tissue.^20,21^ Our model addresses some of the limitations of previous experimental systems by initially forming a 3D tissue-like construct consisting of human fibroblasts and endothelial cell networks embedded in a fibrin gel. Wounding of these 3D vascularized tissues leads to rapid contraction of the wound edge, followed by a lag phase of gradual contraction before cells can migrate into the healing area. Interestingly, endothelial cells are not stimulated to sprout into the healing area in our model. Fibroblasts mediate the primary closure event via contraction of the native fibrin matrix and deposition of cell-derived matrix components including fibronectin and collagen III. This process is consistent with our understanding of granulation tissue formation, which depends on fibroblasts to secrete the matrix and contract the wound edge, and also captures a non-angiogenic dynamic for endothelial cells at the wound edge. This deviates from the rapid endothelial migration observed in 2D *in vitro* assays and may be useful to help understand why it can be difficult to stimulate angiogenesis in some human wounds.^4^

Few comparable studies have investigated the ability of cells to heal *in vitro* tissue by the assembly of a provisional matrix without a predefined substrate on which cells crawl over. One recent study^22^ has investigated the provisional matrix assembly in the context of stromal wound healing; however, the closure in these tissues is observed on the order of hours rather than the multi-day healing that we observe in our study. Several key differences between these two models may help to explain why there is a timescale discrepancy, including the composition of the matrix within the tissues formed and the boundary constraints of the tissue. The stromal tissues are formed in a 2 mg/ml collagen tissue,^22^ whereas our model utilizes a softer and less fibrous gel, composed of 2.5 mg/ml fibrin with 0.4 mg/ml of collagen. Additionally, the size of the stromal tissues is not constrained during healing; these tissues are suspended on pillars, allowing for overall compaction of the tissue during closure.^22^ In our system, the boundary constraint of the PDMS mold may play an additional role in altering the dynamics of closure by maintaining a constant tissue size throughout healing. These comparisons implicate the possible role of matrix stiffness and fiber structure and the ability of the tissue to compact as additional variables to investigate their role in healing dynamics in future studies.

The matrix molecules present in this system are representative of the matrix molecules that are normally deposited by stromal cells during the proliferative stages of wound healing.^23^ Numerous *in vivo* studies have characterized the presence of disorganized fibronectin at early stages post-wounding, which are subsequently remodeled into fibrils^24^ and gradually replaced with collagen fibrils over time.^25^ Collagen III has been detected in rat wound as early as 10 h post-wounding at levels higher than that of the surrounding tissues^26^ and plays a role in preventing the overproduction of scar tissue.^27^ Additional studies have demonstrated the replacement of this collagen III matrix with collagen I fibrils, which increases the tensile strength of the wound.^28^ The deposition of cell-derived matrix proteins that occur within culture model is reminiscent of early granulation tissue, although the full complexity of matrix molecules in this model has not yet been investigated. Notably, fibronectin has been shown to support and promote angiogenesis,^29^ but in our model, the cell-derived matrix alone does not appear to be sufficient to promote angiogenic invasion of the endothelial cells into the newly deposited matrix.

We do, however, observe a contraction of the initial fibrin matrix at the wound edge, which may bring vessels closer to the healing region. This observation is reminiscent of an early hypothesis in the field that suggested that tension at the wound edge could play an essential role in orienting vessels during wound angiogenesis, as described in the context of chorioallantoic membrane and dermal wound healing.^30^ A more recent study supported this hypothesis, observing that translocation of preexisting vasculature during wound healing contributed to the formation of early granulation tissue through a force-dependent mechanical contraction of the wound edge.^31^ The observed contraction of the initial fibrin matrix within our model could indicate a similar role for overall tissue contraction bringing vessels toward the wound center in our model as well.

This contraction-mediated process is distinct from angiogenic vessel ingrowth, which would require the expansion of endothelial cells and the growth of new vessels into the wound, and is understood to be critical to granulation tissue vascularization.^25,32^ Interestingly, our model utilizes a coculture of endothelial cells and fibroblasts, which can spontaneously self-assemble into a robust vascular network within an exogenous fibrin matrix; however, the endothelial cells do not remodel and sprout into the healing region after injury. Thus, while fibroblasts are sufficient to initiate vasculogenesis, other factors may be required to drive angiogenesis into the healed region of our model system.

*In vivo*, many factors and biophysical cues play a role in angiogenesis. Gradients of growth factors including FGF, VEGF, Ang2, and PDGF^32^ can stimulate new angiogenic processes from stable vessels.^33^ Immune cells such as macrophages have been shown to stimulate neovascularization^34^ and promote angiogenesis through “switch-like” behavior associated with the release of growth factors, matrix metalloproteinases, and other matrix modifying enzymes.^35–38^ Blood flow is an additional biophysical factor that activates signaling cascades in endothelial cells that are essential for regulating endothelial cell migration, angiogenesis,^39^ lumen formation,^40^ and vascular remodeling.^41^ Given that all these factors simultaneously regulate sprouting angiogenesis during healing *in vivo*, we propose that our model could be a useful bottom-up system to investigate the minimum components required for appropriate wound angiogenesis.

## CONCLUSION

In this study, we developed a novel model of *in vitro* wound closure that depends on contraction and granulation tissue formation in a three-dimensional, engineered vascularized human tissue. Our findings, thus far, reinforce the importance of fibroblast-mediated contraction of the wound edge and secretion of extracellular matrix molecules to wound closure and allow for real-time observation of blood vessel dynamics during the early phases of granulation tissue development. Further advancements on the model to increase the complexity relative to physiological healing will allow for precise investigations into the molecular and biomechanical factors that play a role in the regulation and dysregulation of angiogenesis during granulation tissue development.

## METHODS

### Cell culture

Human umbilical vein endothelial cells (HUVECs, pooled from 4 donors, Lonza) were cultured in endothelial cell growth medium (EGM-2, Lonza) and used before passage 7. Normal human lung fibroblasts (HLFs, Lonza) were cultured in fibroblast growth medium (FGM-2, Lonza) and used before passage 8. All cells were cultured in a humidified incubator at 37 °C with 5% CO_2_.

### Lentiviral transduction

LifeAct-mRuby-HUVECs were generated using pLenti.PGK.LifeAct-Ruby.W (Addgene plasmid #51009, gift from Rusty Lansford). Histone labeled cells were generated using pRRL.H2B-GFP and pRRL.H2B-mCherry lentiviral plasmids. Lentivirus was produced by co-transfecting HEK 293 T cells with each individual lentiviral plasmid and with the pVSVG, pRSV-REV, and pMDLg/pRRE packaging plasmids using a calcium phosphate transfection method. The virus-containing supernatant was collected 48 h after transfection, concentrated using PEG-it Virus Precipitation Solution (SBI), resuspended in PBS, and flash frozen at −80 °C. HUVECs and HLFs were infected with lentiviral constructs in growth medium for 16–20 h and then cultured in normal growth medium. Lentiviral titers were determined by monitoring the cell growth rate, morphology, and expression levels compared to non-infected control cells.

### Device fabrication

The mold, modified from a previously published study,^8^ was formed using soft lithography. Polydimethylsiloxane [PDMS, Sylgard 184, Dow-Corning)] was mixed at a standard ratio of 1:10 (PDMS base to cross-linking reagent) and then was added to a mold (Protolabs) and cured in a 60 °C oven overnight. Cured PDMS was cut to isolate molds, a 3 mm biopsy punch was used to open the central gel region to the air for wounding, and then, the mold was bonded to cover glass (VWR) by activation of the surface via plasma treatment for 30 s. Bonded devices were incubated in a 100 °C oven overnight. On the day prior to seeding, the PDMS surface was functionalized to increase gel adhesion by plasma treating the molds for 30 s and then adding them into a desiccator with a few drops of (3-Glycidyloxypropyl)trimethoxysilane and attaching the dessicator to house vacuum overnight. On the day of seeding, the PDMS molds were soaked in 70% EtOH for 1 h to remove any excess silane and then UV sterilized for 15 min prior to seeding cells.

### Formation of capillary beds

Both HUVECs and HLFs were lifted from the tissue culture plates with 0.05% Trypsin-EDTA and centrifuged at 200 g for 4 min. Cells were, then, resuspended to a concentration of 25 million cell/ml in EGM-2. Cell suspensions were mixed in individual tubes. The final cell concentration in all tissues was 10 million cells/ml in the tissue, with varying ratios of HUVECs and HLFs, as specified per figure. For all experiments, cells were mixed into a bulk gel composed of fibrinogen (Fibrinogen from Bovine Plasma, Sigma) at a final concentration of 2.5 mg/ml and collagen (type 1, from rat tail Corning) at a final concentration of 0.4 mg/ml. The collagen solution was prepared on ice. Briefly, collagen type I was buffered with 10× DMEM and brought to a pH of 7.7 using NaOH and diluted to a concentration of 2.5 mg/ml with PBS. In experiments where fluorescent fibrinogen is used, 0.2 mg/ml of fluorescent fibrinogen (Fibrinogen from human plasma, Alexa Flour conjugates, 488 and 647, ThermoFisher) was substituted for the same amount of unlabeled fibrinogen. To form tissues, the fibrinogen and collagen solutions were added in rapid succession to the individual tubes containing the cell suspensions. With one tube at a time, thrombin (Sigma) was added for a final concentration of 1 U/ml, and the mixtures were immediately injected into the molds. Gels were polymerized at RT for 2 min, at which point EGM-2 media was added to the reservoirs, and tissues were added to the cell incubator for 15 min. After 15 min, additional EGM-2 media was added directly on top of the gel region, and tissues were returned to the incubator. On each subsequent day, media on the device was aspirated and replaced with fresh EGM-2.

Once formed into tissues, both cell types were cultured in EGM2. We did observe a qualitative decrease in fibroblast contractility upon culture in EGM2, fibroblast tissues formed in FGM-2 contracted extensively and pulled away from the PDMS mold, whereas fibroblasts cultured in EGM-2 did not [Figs. 7(a) and 7(b) in the supplementary material]. Fibroblasts expanded normally in either media when cultured in 2D, and Western blotting for aSMA and pMLC did not differ significantly in either media [Fig. 7(c) in the supplementary material].

### Tissue wounding

After three days of culture, vessel networks had self-assembled within the three-dimensional tissue. These tissues were, then, wounded with a full-thickness incision by using a diamond dissection knife (ME122, Electron Microscopy Systems, #72029), which was controlled using an XYZ micromanipulator (SLC-2040, SmarAct GmbH). Tissues were mounted over a Nikon TE200 brightfield microscope with a 10× objective in order to monitor the wounding progress. These tissues were cut in layers until a full-thickness wound was visible.

### Bulk gel rheology

The rheological properties of the bulk gel were determined using a TA Instruments DHR-2 Rheometer. A 2.0 mg/ml solution of collagen I at pH 7.7 was mixed with a solution of fibrinogen to yield a final solution of 2.5 mg/ml fibrinogen and 0.4 mg/ml collagen I. Thrombin was added at a final concentration of 1 U/ml, and the solution was mixed and rapidly added to a preheated 20 mm parallel plate geometry. The plate was lowered to a 1 mm gap, and the solution was left to gel for 30 min prior to the measurement. Storage and loss modulus were determined from a frequency sweep between 0.01 and 10 Hz with a 1% strain, and reported values for each condition are the average of 5 measurements at equivalent frequencies per sample.

### Quantification of 3D cell migration

Tissue healing was monitored using a microscope per day. Non-fluorescent cells were monitored via brightfield imaging daily using a Nikon TE 200 microscope with a Nikon 10× objective and Spot Imaging 5.3 software. Tissues containing fluorescent cells were imaged immediately after wounding and on subsequent days with 150 *μ*m stacks of images on a Yokogawa CSU-21-Zeiss Axiovert 200M inverted spinning-disk microscope using a Zeiss 10x/0.45NA air objective and an Evolve EMCCD camera (Photometrics) inside of a 37 °C heated environmental chamber. Quantification of cell migration was performed using ImageJ.^42^ Images of each cell type were individually projected onto a single image via the “max intensity” projection with caution used to exclude the bottom glass from these projections in order to avoid quantification of cells migrating on 2D glass. The background was subtracted, and a threshold was applied to the image to isolate nuclei. An ellipse was fit to the shape of the wound on day 0. This ellipse was overlaid onto the images of subsequent days and applied as a mask, isolating cells within this region. The “watershed” command was, then, used to separate overlapping nuclei, and the “analyze particles” command counted the nuclei per frame.

### Time lapse imaging

For continuous imaging at multiple time points, imaging was performed with a Nikon Ti Eclipse (Nikon Instruments, Inc.) microscope, which utilized an Evolve 16-bit electron-multiplying charge-coupled device camera (Photometrics) with an A-Plan 10× objective, all contained within a humidified chamber maintained at 37 °C and 5% CO_2_. For the 24 h time lapse, bright field images were captured every 30 min over the course of 24 h. Wound areas were quantified using the ImageJ measurement tool from outlines of the wound edge. For the multi-day time course, bright field and fluorescent images were captured once per hour for 96 h. EGM-2 media for these experiments was supplemented with OxyFluor (Fisher) at a 1:100 dilution to prevent phototoxicity in the cells.

### Quantifying direction of cell migration

To quantify cell migration, the ImageJ plugin “Trackmate” was utilized. A region of interest was chosen surrounding the wound, and nuclei were automatically isolated and tracked using the LAP tracker option, generating information about individual cell tracks. This output file from the Trackmate algorithm was read into Matlab^®^ (Mathworks) to generate plots. In Matlab, the endpoints of each track were used to calculate net displacements over 6 h and to calculate the angles of each net displacement over the 6 h period.

### Tracking of the fibrin matrix

The channel representing the fluorescent fibrin matrix was isolated from the 150 *μ*m stacks of multidimensional images obtained during cell tracking. ImageJ was used to Z-project the maximum signals of the fibrin matrix into a 2D plane. A threshold was applied to this image to isolate the void in the matrix where the original wound existed. The non-zero area representing the area devoid of the fibrin signal was quantified using the ImageJ measurement tool.

### Immunofluorescence

Tissues were fixed in 4% paraformaldehyde in PBS containing calcium and magnesium for 20 min and blocked with a solution of 2% bovine serum albumin (BSA, Sigma) dissolved in PBS overnight on a rocker (Benchmark Scientific) at 4 °C. All antibodies were diluted in blocking solution and applied overnight on the rocker at 4 °C. Endothelial vessels were imaged using Lectin (UEA DyLight 649, Vector Labs), diluted 1:300 in blocking solution. Antibodies against fibronectin (ab26245, 1:100) and collagen III (ab7778, 1:100) were purchased from Abcam. All IgG secondary antibodies (Alexa Fluor 488, 568, and 647 goat anti-rabbit and goat anti-mouse) were purchased from Thermofisher Scientific. Matrix staining was imaged on a Yokogawa CSU-21-Zeiss Axiovert 200M inverted spinning-disk microscope using either a Zeiss 20× air or 40× water objective and an Evolve EMCCD camera (Photometrics). Identical laser intensities and microscopy settings were used per objective across all samples. The immunofluorescence intensity of the matrix was quantified from 5 to 6 identical regions of interest per image for both fibronectin and collagen III and was normalized to the average intensity of the control tissue image. Staining of the wound for 3D visualization was imaged on a Leica SP8 confocal microscope (Leica, Wetzlar, Germany) using either a Leica 10x/0.30 NA water objective and the Leica LAS X imaging software.

### Ethics Approval

No ethics approval was required for the experiments described in this study.

## SUPPLEMENTARY MATERIAL

See the supplementary material for figures and movies of the wound healing process.

## Data Availability

The data that support the findings of this study are available upon reasonable request from the corresponding authors.
